# Notes and News

**Published:** 1888-09-22

**Authors:** 


### Notes and News.

Collected and Communicated.

The Victoria Hospital at Burnley, which was opened a year or two ago by Prince Albert Victor with 44 beds, has recently been enlarged to accommodate 52 in-patients.

As the result of the recent appeal made by the London Hospital on behalf of its maintenance fund, the sum of £43,509 has been brought into the treasury.

The swallows, it is said, are rapidly mobilising for their southern journey. Weather prophets hence infer an early winter.

Mr. Capel Carter, formerly of Woodford, but late of Bath, bequeathed £3,000 to the London Hospital, £3,000 to the Charing Cross Hospital, and £2,000 to the Dental Hospital of London.

On Wednesday, September 12th, the Council of University College, Dundee, appointed Mr. Patrick Geddes, of Edinburgh, to be Professor of Botany, and Mr. Andrew Paterson, lecturer at Owens College, Manchester, to be Professor of Anatomy.

The allotments question is advancing apace throughout the country, to the great advantage of both urban and rural labourers. The Rector of Stockton, near Rugby (Rev. W. Tuckwell), is a strong advocate of fixity of tenure, and has an influential following on that crucial point.

A large additional ward has been added to the accommodation of the Consumption Hospital, Thronemount, Belfast, giving increased space, which was very urgently needed, the number of applications for admission being at all times far in excess of the capacities of the institution.

Asylums for the insane are no longer considered such perfect models of applied science as they were some time ago held to be. A recent correspondence in the *Times* has brought to light many facts and opinions which point the way to more intelligent treatment for those who are mentally deranged. There can be no doubt that many cases of insanity should be treated not in an asylum at all, but in a hospital specially adapted for that purpose. At present no such hospital is to be found; but if any form of disease can justify the existence of special hospitals, it is the temporary and curable cases of mental disorder.

"With reference to the missing parts of Annie Chapman's body," says the *Star*, "Thomas Bolas writes: 'That biologists have been so infatuated by their pursuits as to cause murder to be committed in aid of their researches is a matter of history, and to my mind there is quite enough evidence of the last murder being the work of some half-mad physiologist in search of living tissues or organs from a healthy subject, for experiments on graftation, to justify certain investigations by the police.'" The *Star* makes no comment. We will be less reticent, and express the opinion that Thomas Bolas is a fool.

The National Coffee Tavern Conference at Hull, which has just been held, is urgent to bring about a reform in the drinking habits of the community. It has been suggested that the different companies shall unitedly offer a prize of £2,000 for a national, non-intoxicating drink to take the place of beer.

The Mildmay Home for patients in advanced consumption has published its annual report, which shows that after paying all demands, there is a balance in favour of the institution of nearly £200. The Home is situated at Torquay, and the honorary secretary is Mr. F. Gumbleton, of Connemara, Torquay.

It is said that at Stratford-on-Avon the Church of England is the only religious body which contributes anything to the local hospital. It is difficult to believe that this can be true. Perhaps there is some local reason for it. If there be, the Noncomformists should publish it if they wish to escape the charge of meanly shirking one of the most elementary of Christian duties.

The eighth report of the Inspector of Retreats, under the Habitual Drunkards' Act, 1879, for the year 1887, recently published, shows that sixty-six persons in the aggregate were admitted to the seven retreats during the year, as against seventy-three in the previous year. Several patients obtained their discharge for various reasons before the usual time. Very satisfactory work was reported from all the retreats.

"As regards vegetable diet," says a well-known medical man, "I speak from experience, for I have tried it for many weeks. I found it made absolutely no difference to my health, strength, and spirits; but I confess I was glad to return to the fleshpots. I am inclined to think that was the voice of true wisdom which commanded Peter to 'kill and eat all sorts of four-footed beasts of the earth, and wild beasts, and creeping things, and fowls of the air.'"

In consequence of the numerous suicides in Liverpool by the drinking of carbolic acid, the health authorities of that city have determined to memorialise the Privy Council with the view of having the sale of carbolic acid placed under restrictions similar to those regulating the sale of other poisons. This somewhat tardy action on the part of the Liverpool authorities calls public attention to a defect in the law which should have been remedied long ago.

The industrial department of the Girls' Friendly Society held a conference at Harrogate last month, under the patronage of Lady Louisa Lascelles, Mrs. Fawkes, of Farnley Hall, Otley, and Mrs. Jerome Mercier, of Kemerton. It was decided to offer prizes for the best-made butter, cheese, bread, and cakes, the members to compete at diocesan or branch festivals. The object is to induce country girls to revive that practical form of their education which has recently been too much neglected, to the injury of the farming interest.

Dr. Francis Sutherland states that "in London the abuse of hospital privileges is carried to a further length than in any other city in the kingdom. . . . It would be impossible to improve upon the facilities afforded by the present system, which admits free many who could afford to pay. . . . One certain result of a central administration would be that people with adequate means would find it extremely difficult to get into the wards, as there would be—what there is not a semblance of at present—an inquiry made into their position. Moreover, there is great need for a central bureau for laying down without fear or favour some general indications as to what diseases are suitable for hospital treatment, and what class of people should have free access. The state of the London hospitals must sooner or later bring the hospital question to a head."

THE following vacancies are announced this week, the amount of salary in each case being given after the name of the institution :—

**Resident Medical Officers.**

East London Hospital for Children, Shadwell, E. Resident Clinical Assistant.—Board ; no salary.
Fulham Union. Assistant Medical Superintendent.—£100 per annum, with board, etc.
Great Northern Central Hospital, Holloway Road, N. House Physician.—£50 per annum, with board, etc.
North Shields and Tynemouth Dispensary. House Surgeon and Dispenser.—£130 per annum, with furnished house.
St. Marylebone General Dispensary, Welbeck Street, W. Resident Medical Officer.—£105 per annum.
Westminster General Dispensary, Gerrard Street, Soho. House Surgeon.—£100 per annum.

**Non-resident Medical Officers.**

City of Liverpool. City Hospital, Park Hill, and City Hospital, Grafton Street. Visiting Physician.—£100 per annum. Candidate must be University Graduate, or M. or F.R.C.S.
Dental Hospital of London, Leicester Square. Lecturer on Dental Surgery and Pathology.
Great Northern Central Hospital, Holloway Road, N. Physician. Candidates must be M.B. or M.D., and F. or M.R.C.P.
Westminster Hospital, S.W. Fourth Assistant Physician.
Westminster Hospital, S.W. Administrator of Anæsthetics.

A SCHOOL of Sanitary Plumbing has lately been established by the Council of the Association of Working Plumbers in the Department of the Seine, for the purpose of giving these artisans better instruction in the practical part of their trade, and a competent knowledge of the principles of hygiene relating to their work. Besides technical courses, Dr. A. J. Martin will lecture on the hygiene of the dwelling, and M. L. Masson on sanitary plumbing.

DR. DAVID ROXBURGH, in his annual report to the managers of the Isle of Man Hospital, calls attention to the fact that the increase in the number of patients during the year is to some extent due to the increasing abuse of the charity by those who are able to pay for their medical and surgical advice. This is more especially the case with casualties, where the difficulty of deciding who are and who are not proper patients is increased by the necessity and urgency of the cases.

HOSPITAL and surgical nurses may obtain a practical demonstration of very great value by paying a visit to the Irish Exhibition. Mr. McIlroy, who is deservedly famous for a great variety of surgical beds, chairs, tables, etc., exhibits several of his principal constructions, and gives most intelligent and interesting accounts of them. His stand will be found near the refreshment-room at the northwest corner. A more intelligent knowledge of the positions of the body and other essentials of certain departments of surgery may be obtained in half an-hour from Mr. McIlroy than will ordinarily be picked up in a twelvemonth in the hospital. Senior nurses especially should make a point of visiting these exhibits.

THE mechanical restraint of the insane is a subject deeply interesting to every one, inasmuch as every man, woman, and child born into the world is a potential lunatic. A correspondent in the *Times* writes : "In an asylum containing less than three hundred patients, there have been thirty-eight instances of mechanical restraint in twenty-seven days. . . . If this had taken place in a private asylum, the outburst of public indignation would have compelled the immediate closure of the house. . . . . It happens, however, in the premier institution in Britain, in a royal hospital of unbounded prestige and unlimited resources, in an asylum to which the most difficult cases are refused admission. This state of things, which would be a disgrace to the officers of a workhouse, happens in the model institution to which the profession throughout the country looks for light, leading, and example ; and yet the medical officer has not a word to say in extenuation, and the presiding officer, Sir James Clarke Lawrence, does not consider it worth investigation."

THE Infirmary Ball at Ryde appears to have been a failure. One critic is of opinion that the unfortunate circumstance was due to the fact that it was known beforehand there would be a bad supper. Another refuses to believe that those "epicureans who attend balls, not to enjoy a dance, but to guzzle viands washed down with champagne," knew anything at all about the matter. Be that as it may, for some reason the attendance was thin, and the result to the Infirmary anything but satisfactory.

FRANCES MADDEN writes to the *Daily Chronicle* to the effect that she has been refused admission to the Nursing Department of St. Thomas's Hospital because of her religion. At first she was most favourably received, and all continued to go well until she avowed herself a Roman Catholic. "We are all Protestants here, and that ends the matter." That is one side of the question. We can express no opinion, and we should advise the public to observe the same neutral attitude until the other side has been heard.

"LARGE as the infirmary (Blackburn) is," says a local contemporary, "it yet lacks many conveniences. We did not notice any common-room for the nurses and servants when off duty. The bedroom accommodation for the servants is by no means perfect. Where do they all get stowed away? Some of them find a lodgment in the cellars. This is not as it should be. The health upon which the spirits (and efficiency) of nurses are so dependant is a prime consideration. When they are weary and tired they should have other places than their bedrooms, where they may enjoy some of the benefits of the family circle, and of a free social intercourse." It is to be feared that there are very many hospitals and infirmaries, both in town and country, to which this description would apply ; and perhaps this is not to be wondered at, seeing that the chief consideration which naturally guides hospital managers is the care and cure of the sick inmates. But even from the point of view of the poor sick and the subscribers, it is a real economy of strength and money to treat attendants well. Besides, it is surely a very barbarous and short-sighted policy to kill nurses in order to cure patients.

THAT large portion of the public who read with pleasure the popular science of the times, will have heard with deep regret and a feeling amounting to pain, of the sudden death of Mr. Richard A. Proctor. Mr. Proctor left his Florida farm apparently quite well, and arrived in New York on Monday week, intending to sail for England last Saturday. That Saturday he never saw. On leaving the Westminster Hotel, New York, he felt ill, but thought the feeling was produced by the fatigue of the journey. Next morning he was much worse, and a doctor was summoned, who, finding alarming indications, called in a consultant. The symytons of yellow fever were present, and it was decided to send Mr. Proctor to the hospital. On Wednesday afternoon the dreaded "black vomit" made its appearance, and there was no longer any room for doubt. At a quarter past seven the same day life was over. Mr. Proctor remained cheerful and hopeful almost to the last. Those who were accustomed to read Mr. Proctor's writings – and they were a very large number—looked upon him rather in the light of a trusted scientific guide and friend, than a mere writer. His enthusiasm for knowledge was great, but his love of communicating it was greater still. By stupid Dryasdusts he was regarded with that kind of suspicion which plodding dulness always entertains for bright and active genius. If a man be brilliant he may be scientific, if he be stupid he must be, is the first article of many people's scientific credo. Mr. Proctor was both brilliant, scientific, and human—propositions which are true of very few scientific men. His loss will be deeply and universally mourned.

